# Serial Mediation Models of Future Anxiety and Italian Young Adults Psychological Distress: The Role of Intolerance of Uncertainty and Non-Pathological Worry

**DOI:** 10.3390/ejihpe14060121

**Published:** 2024-06-20

**Authors:** Giorgio Maria Regnoli, Gioia Tiano, Barbara De Rosa

**Affiliations:** Department of Humanities, University of Naples Federico II, Via Porta di Massa 1, 80133 Naples, Italy; giorgiomaria.regnoli@unina.it (G.M.R.); gioiatiano@gmail.com (G.T.)

**Keywords:** anxiety, depression, future anxiety, intolerance of uncertainty, mental health, non-pathological worry, serial mediation analyses, stress, young adults

## Abstract

Previous research has already examined the relationship between Future Anxiety, a construct recently introduced in Italy, and mental health in young adults, although possible mediating variables in this relationship have so far never been investigated. The present study attempts to fill this gap by exploring the incidence of Future Anxiety on psychological distress (i.e., Stress, Anxiety and Depression) in a group of 302 young Italian adults (18–30 years; *M* = 21.9; *SD* = 2.6; 49.0% males; 51.0% females), presenting and evaluating the simultaneous mediating effect of Intolerance of Uncertainty and Non-Pathological Worry. Findings highlighted how Future Anxiety had a positive and significant direct effect on Stress and Depression, but not on Anxiety. In the three serial mediation models proposed, Intolerance of Uncertainty and Non-Pathological Worry mediated the relationship between Future Anxiety and mental health outcomes. The results also confirmed the hypothesized serial mediation effect by highlighting how young adults with greater Future Anxiety experienced more Intolerance of Uncertainty, which positively affected Non-Pathological Worry levels and, in turn, exacerbated psychological distress. Finally, results indicated that female participants experienced more Stress, Anxiety, and Depression in relation to Future Anxiety compared to males. Starting from the review of main references on this subject, the results discussed provide new insights for understanding youth psychological distress. Finally, practical implications for the design of supportive interventions for this study’s target group are proposed.

## 1. Introduction

For more than a decade, young adulthood has come to the forefront of psychological literature due to the complexity of its developmental tasks, exacerbated by economic and social difficulties as well as cultural characteristics that are typical of contemporary Western societies [1,2,3,4,5]. As an effect of this intertwining of factors, the time dilation of developmental tasks and acquisitions seems to have become the hallmark of this transitional phase [6]. In the Italian context, the specific subject of this research, procrastination is the central marker of the transition to adulthood, whether it concerns access to the world of work, a stable relationship, or parenthood [7,8]. Well beyond Italian borders, it has been highlighted that the tendency to procrastinate fuels experiences of frustration, bewilderment, and inadequacy [9,10,11], with negative repercussions on self-esteem, the representation of the future and, in general, on the mental health of young adults [12,13,14].

The worsening of psychological well-being and the growth of syndromes and symptoms of the internalizing and externalizing sphere occurs particularly in women [15,16], and has been exacerbated by the COVID-19 pandemic, which left deep marks on this developmental target, as reported in studies conducted in several countries [17,18,19,20,21]. If the pandemic trauma has slatentized and potentiated prior psychological distress [15,19,22,23,24,25], the ‘youth mental health emergency’ that is being talked about in Italy [26] urges psychological research to not only deal with it but to understand how to better cope with it, which in our opinion is a necessary step to investigate the risk and protective factors that may affect it.

Previous literature shows that maintaining a positive perspective of the future in the transition to adulthood is a protective factor that supports the process of identity definition, planning, emotional regulation, and the management of states of uncertainty [1,27,28,29,30]. The ability to preserve hope for the future, despite the inevitable uncertainties associated with potential obstacles and difficulties, helps young adults to maintain positive personal and professional expectations [31,32]. However, nowadays, the increasing difficulties in finding stable employment positions, reaching economic autonomy, and leaving one’s family home [7,33] have fueled a negative representation of the future in young people [34,35,36]. This socio-economic precariousness has been compounded by traumatic or potentially traumatic collective events such as the pandemic, wars, and the climate crisis, an interweaving of factors that has increased worries about the future of the world and one’s destiny, pessimism towards the future, and a deep sense of uncertainty, making it increasingly distressing to ‘think of oneself as an adult’ [37,38].

Recently, Jannini et al. [39] introduced the construct of Future Anxiety, originally defined as an attitude toward the future in which negative cognitive and emotional processes dominate over positive ones and where fear prevails over hope [40]. The negative time perspective and the consequent pessimism are not only powered by difficulties in the surrounding reality but also by a perceived personal inability to cope with them [39]. In fact, according to Zaleski [40], life experiences and ways of reacting to fear and worry were to be understood as predisposing factors to Future Anxiety. At the same time, this construct stems from the need to investigate the impact that the worsening of global social, economic, and political conditions is having on mental health [41], since the vision of the future is also strongly influenced by contextual factors.

Future perspective and the temporal dimension become particularly important in adolescence when the acquisition of increasingly complex cognitive skills—such as, for example, hypothetical-abstract thinking—enable a less abstract conception of time, which thus assumes a significant role in defining personal identity [42,43]. In a life span perspective, Susulowska [44] highlighted how fear of the future appeared precisely between the ages of 11 and 14 but that its frequency and intensity increased between the ages of 15 and 19 but it peaked between the ages of 20 and 29.

Recently, in a study by Mutia and Hargiana [45], Future Anxiety appears to apply specifically to young adulthood, and contextual factors have a greater role in facilitating or hindering the growth process. In contrast, however, Awad et al. [46] highlighted how significantly lower levels of Future Anxiety were found with advancing age, further highlighting the construct’s centrality in young adulthood.

In a study conducted in the Italian context [39], Future Anxiety showed positive and significant associations with depression and anxiety as well as with irascible and anxious temperament and neuroticism. Similarly, research carried out in other cultural contexts is in line with the above-mentioned findings and confirms the positive and significant association between Future Anxiety and psychological distress (stress, anxiety, and depression) while also highlighting significant gender differences in Future Anxiety levels [46,47]. Recent research has also explored the relationship between Future Anxiety, collective phenomena with trauma potential (e.g., pandemic, war) and psychological distress and highlighted its function as a risk factor in exacerbating the impact of such events on mental health [20,48,49,50].

States of apprehension and worry about the future that increase fear of the unknown and, at the same time, a negative attitude towards the future [41], facilitate the arising of experiences of uncertainty that risk enhancing the effect of a physiological instability that is typical of the transition to adulthood. Indeed, the construction of identity as a specific developmental task is linked to the exploration of the various possibilities that the present and future might offer, but which have not yet materialized [2]. Thus, while the ability to tolerate and manage uncertainty fosters exploration by acting as a protective factor for well-being [32], the inability to tolerate it constitutes a risk factor, increasing anxiety and reverberating negatively on developmental tasks [51]. Intolerance of Uncertainty is described as a dispositional component that, in the interplay between cognition, emotion, and behavior, expresses “the tendency to be bothered or upset by the (as yet) unknown elements of a situation, whether the possible outcome is negative or not” [52] (p. 6). Research has shown that this dispositional component can fuel anxiety, fear, and worry [53], influencing how individuals interpret the present and future [54] and facilitating the adoption of dysfunctional behaviors in service of an illusory desire for control [52]. Moreover, other studies have shown how it constitutes a trans-diagnostic factor for psychopathological frameworks of the internalizing sphere, including obsessive compulsive disorder, generalized anxiety disorder, depression, and eating disorders [55]. More recently, it has been found that Intolerance of Uncertainty can not only be powered by stressful external events—such as a pandemic or war—but that it also enhances the impact of such events on mental health, as does Future Anxiety [49,56]. Recently, Yang et al. [57] explored the relationship between future perspective and Intolerance of Uncertainty and found that more future-oriented people were able to set long-term goals more easily and had greater self-control as well as lower levels of Intolerance of Uncertainty. Having a dark and anxious view about the future, on the contrary, could contribute to feeding an intolerance toward states of uncertainty exacerbated by the possibility that negative events, uncontrollable in the present, may occur in the future [40]. The relationship between future vision and Intolerance of Uncertainty has also been explored by Carleton et al. [54] who, conceptualizing the dimension of “Prospective Intolerance of Uncertainty”, shed light on how the inability to control threatening future events or situations can cause in some individuals an increased intolerance toward states of uncertainty and ambiguity.

Within the Italian framework of the ‘youth mental health emergency’, a sharp increase in worries associated with both global collective phenomena such as the climate crisis and more generally the future, as well as strictly contextual issues related to unemployment, the economic crisis, and the rising cost of living have already been noted [37,58]. The relationship between worries and mental health has long been investigated in the literature [59] and concerns both pathological worries and non-pathological worries. Indeed, while the former are associated with lower psychological well-being [60,61], the latter—viz. daily worries triggered by everyday stressors [62,63]—are also capable of increasing loneliness, dissatisfaction with life, and forms of mental suffering such as anxiety and stress [64,65,66]. Thus, the Worry construct is significantly related to mental health outcomes, but it is also associated with Future Anxiety [40]. Despite their differences, the link between the two constructs is clearer insofar as the latter is described as the tendency to think about the future with particular worries, fears, and anxieties [39]. Indeed, the relationship between worry and perception of the future has been noted in the past by MacLeod et al. [67] who conceived of worry precisely as a cognitive process related to future events with uncertain outcomes that may also be accompanied by anxiety. Tallis et al. [68] shed light on how the perception of threatening events and/or situations in the future is a central element in triggering the worry process. According with this contribution, Bentz and Williamson’s study [69] found that the perception of a greater or lesser likelihood of threatening events occurring in the future was particularly significant in predicting higher levels of anxiety and worry. The findings of the above-mentioned contributions showed how the future perception of threatening and/or uncertain situations—a construct in some ways similar to the conceptualization of Future Anxiety [39,41]—plays a pivotal role in the Worry cognitive process. Therefore, these contributions oriented the association hypotheses between the variables in our study. 

Furthermore, since the publication of Freeston et al. [70], the relationship between Intolerance of Uncertainty and Worry has also been extensively investigated, becoming an important constituent axis of the Intolerance of Uncertainty Model (IUM) [71]. In this vein, Koerner and Dugas’ study [72] showed that Intolerance of Uncertainty can be considered a cognitive vulnerability factor for Worry, as it can significantly predict increased levels of Worry both in a non-clinical sample [73] and in an experimental research setting [74]. To sum it up, scientific evidence suggests that Intolerance of Uncertainty is a risk factor that exacerbates overall Worry levels by reinforcing a negative problem orientation and fostering cognitive avoidance strategies [71]. From a neurobiological perspective, Grupe and Nitschke [75] further highlighted the interconnection between Intolerance of Uncertainty and Worry as well as between future anticipation of events and Intolerance of Uncertainty. They detected specific activations of the insula, amygdala, and cingulate cortex involved, respectively, in the pre-vision of how one would feel in response to specific outcomes, in attention focusing on threatening events as well as on the anticipation of future adverse events. In the activation of these brain areas, the ventromedial prefrontal cortex (vmPFC) plays an important regulatory function, with direct consequences on greater or minor tolerance toward states of uncertainty [76]. The regulatory function of the vmPFC allows, in individuals without any psychopathologies, the selection of functional coping strategies for managing states of uncertainty such as, for instance, seeking environmental information which can diminish the threatening feeling of uncertainty and facilitate the prediction of future negative events. These brain areas, and, in particular, the ventral and dorsal prefrontal cortexes, also play a key role in the adaptive regulation of worry [77,78] and these neurobiological correlates, some of them overlapping, further confirm the strong interconnection between Uncertainty Intolerance and Worry [71,72,73].

### Aim and Hypotheses of the Study

Based on the literature review, the present study explores the impact of Future Anxiety on psychological distress in a group of young Italian adults, taking into consideration the relationships evidenced therein between the previously described constructs [40,46,53,55,65,68,71,72] and the results of the reports on the mental health status of Italian youth [26,37,79]. Furthermore, it explores the possible joint mediating role of Intolerance of Uncertainty and Non-Pathological Worry. The research aim arises from the desire to fill a gap concerning the absence of mediation studies on the relationship between Future Anxiety and Psychological Distress. At the same time, this study was designed to explore the underlying factors of youth psychological distress—which is growing exponentially in Italy [26]—trace its contours, and highlight potential risk and protection factors that may affect it. In our opinion, this goal can implement the understanding of the long-reported state of ‘youth psychological emergency’, providing potential evidence to support psychological interventions aimed at this age group. 

**Figure 1:**
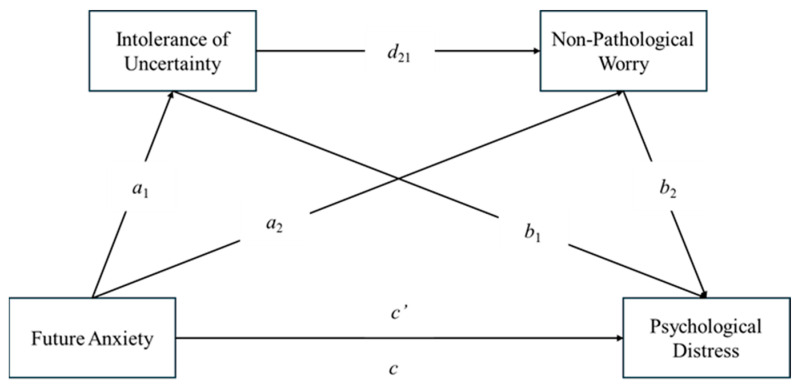
Future Anxiety: predictor variable (X); Intolerance of Uncertainty: Mediator 1; Non-Pathological Worry: Mediator 2; Psychological Distress (Stress, Anxiety and Depression): outcome variables (Y); *c*’: direct effect of Future Anxiety on Psychological Distress (H_2_); *a*_1_: effect of Future Anxiety on Intolerance of Uncertainty (H_3_); *a_2_*: effect of Future Anxiety on Non-Pathological Worry (H_4_); *b*_1_: effect of Intolerance of Uncertainty on Psychological Distress (H_5_); *b*_2_: effect of Non-Pathological Worry on Psychological Distress (H_6_); *d*_21_: sequential double mediating effects on the relationship between Intolerance of Uncertainty and Non-Pathological Worry (H_7_); *c*: total effect of Future Anxiety and Mediators on Psychological Distress.

From the previously mentioned literature, we hypothesized that Future Anxiety would be positively associated with Intolerance of Uncertainty and Non-Pathological Worry, but also with Stress, Anxiety and Depression [H_1_]. We also assumed that Future Anxiety positively predicts Psychological Distress, in particular, Stress, Anxiety and Depression [H_2_] but also that it has an impact both on Intolerance of Uncertainty [H_3_] and Non-Pathological Worry [H_4_]. We further hypothesized that both Intolerance of Uncertainty [H_5_] and Non-Pathological Worry [H_6_] positively predict higher levels of Psychological Distress. Finally, we also assumed that the influence of Future Anxiety on Psychological Distress would be mediated by the joint effects of Intolerance of Uncertainty and Non-Pathological Worry [H_7_], and that women may show higher levels of Psychological Distress than men [H_8_]. The hypotheses are graphically represented in Figure 1.

## 2. Materials and Methods

### 2.1. Procedure and Participants

Participants involved in the study were recruited via social media through the snowball sampling method, in the period between November 2023 and January 2024. Data were collected through self-report questionnaires using the Google Forms platform https://www.google.it/intl/it/forms/about/ accessed on 15 November 2023.

Sampling was performed taking into consideration the following inclusion criteria: age between 18 and 30 years old, residence in Italy, Italian nationality, and acceptance of the informed consent on the first page of the survey. All participants were adequately informed of their right to privacy and anonymity, as well as the aims and procedures of the study.

Two a priori analyses were conducted to define the minimum sample size. G*power program (version 3.1.9.6) was used preliminarily and, by selecting linear multiple regression, fixed model, and *R*^2^ increase, a group of 164 participants was indicated for a medium sample size effect (*f*_2_ = 0.15) with 99% power and an alpha of 0.01. Having adopted the bootstrap method to assess the significance of indirect mediation effects, the MedPower program [80] was used and a minimum sample size of 214 was sufficient to achieve 80% power of detecting a significant effect at the 0.05 level. Thanks to the bootstrapping procedure and our sample size, there are no concerns about sampling power. 

A sample of 302 young Italian adults was recruited for the present study, including 148 males (49.0%) and 154 females (51.0%) aged between 18 and 30 years (*M* = 21.96; *SD* = 2.61). Most participants lived in South Italy (87.1%) and, in particular, 127 young adults declared to live in the city (42.1%) and 175 in provincial areas (57.9%). Concerning marital status, 155 (51.3%) participants were single, 142 (47.0%) were in a non-cohabiting relationship, and 5 (1.7%) were cohabiting with their partners. Regarding their educational level, 8 participants stated that they received a lower secondary school diploma (2.6%), 229 a high school diploma (75.8%), 48 a bachelor’s degree (12.6%), and 27 a master’s degree (8.9%). As for the participants’ occupation, 190 were students (62.9%), 57 were working students (18.9%), 48 were workers (15.9%), and 7 were unemployed (2.3%).

### 2.2. Data Collection Tools

*Personal information*. An ad hoc questionnaire was created to assess participants’ socio-demographic characteristics. Information regarding age, gender, type of residence, region of residence, occupation, level of education, and relationship status were collected.

The *Dark Future Scale* (DFS) [39] is a scale that assesses Future Anxiety through 5 items that explore concern and anxiety about the future, considering the cognitive and emotional processes that induce fear for the future to dominate over hope. This tool is a 7-point Likert type, and the response range is from 0 (Definitely untrue) to 6 (Definitely true). The overall score ranges from 0 to 30, and higher scores indicate higher levels of Future Anxiety. The scale revealed excellent psychometric properties [39] and in this study, Cronbach’s *α* was 0.88.

The *Intolerance of Uncertainty Scale—Short Form* (IUS-12) [54,81] is a 12-item self-report measure with a 5-point Likert-type scale, ranging from 1 (Strongly disagree) to 5 (Strongly agree), assessing one’s tendency to find uncertainty as distressing. The measure provides two different sub-dimensions of intolerance toward uncertainty, “Prospective Intolerance of Uncertainty” and “Inhibitory Intolerance of Uncertainty”. The scale also provides an overall score ranging from 12 to 60 and higher values are indicative of greater Intolerance of Uncertainty [81,82]. In this study, the global tool score was chosen. The original authors report excellent psychometric properties of the tool and good internal consistency [81]. In this study, Cronbach’s *α* for the overall scale was 0.88.

The *Worry Domains Questionnaire* (WDQ) [83,84] is an instrument that assess the Non-Pathological Worry through 25 items grouped in five dimensions: Relationships, Lack of Confidence, Aimless Future, Work-Related, and Financial. This tool is a 5-point Likert-type and response range is from 1 (Not at all) to 5 (Extremely). In addition to five dimensional scores, this instrument also provides a global Non-Pathological Worry score, namely an indication of a widespread and generalized tendency to worry which is not a pathological condition per se, but rather a normal mental activity fueled by everyday stressors. In line with other studies [66,85], we considered the global score here. The Italian adaptation of the WDQ shows good psychometric properties and good internal consistency [84]. In this study, Cronbach’s *α* was 0.92

The *Depression, Anxiety and Stress Scale* (DASS-21) [86,87] is a self-report-instrument useful assessing Stress, Anxiety and Depression in the genal (i.e., nonclinical) population. The tool is a 4-point Likert-type scale, consists of 21-item, grouped into three subscales (7 items for each dimension), exploring self-reported levels of Depression, Anxiety and Stress in the last 7 days. The range for each statement goes from 0 (Did not apply to me at all) to 3 (Applied to me very much, or most of the time). For the Stress dimension, normal levels are recorded with scores between 0 and 10, mild levels with scores between 11 and 18, moderate levels with scores between 19–26, and severe levels with scores between 27 and 34 and extremely severe levels with scores between 35 and 42. As far as the Anxiety dimension is concerned, normal levels are recorded with scores between 0 and 6, mild levels with scores between 7 and 9, moderate levels with scores between 10 and 14, severe levels with scores between 15 and 19, and extremely severe levels with scores between 20 and 42. Finally, for the Depression dimensions normal levels are obtained with scores between 0 and 9, mild levels with scores between 10 and 12, moderate levels with scores between 13 and 20, severe levels with scores between 21 and 27, and extremely severe levels with scores between 28 and 42. The good psychometric properties reported by the Italian authors in the instrument’s adaptation and validation process [87] are also confirmed in this study where Cronbach’s *α* was 0.82 for Stress, 0.88 for Anxiety, and 0.88 for Depression.

### 2.3. Data Analysis

All the analyses were conducted using IBM SPSS version 29.0 [88]. 

Descriptive analyses, mean, minimum and maximum response range and standard deviation were calculated for all psychological variables. Reliability analyses were conducted using Cronbach’s α, which was considered good when values were greater than 0.70. Skewness and Kurtosis were evaluated considering values ranging from −1.5 to +1.5 [89]. 

To explore differences between groups—considering the socio-demographic variables—and highlight possible covariates to be added in the mediation models, *t*-test and ANOVA analyses were performed (*p* < 0.05). Cohen’s d and eta-square (*η*^2^) were used to measure effect sizes.

Correlation analyses were also performed using Pearson’s coefficient (0.10 < *r* > 0.29 = small association; 0.30 < *r* > 0.49 = medium association; *r* > 0.50 = large association; *p* < 0.05) to preliminarily explore the association between variables and to test Hypothesis 1 [H_1_]. 

Before conducting the mediation analyses, preliminary statistical analyses were implemented. To check the common method bias produced by self-report data, Herman’s single factor test was performed [90]. A multicollinearity analysis between the independent variables and the mediators was also conducted. Values of tolerance greater than 0.1 and Variance Inflation Factor (VIF) smaller than 5.0 were considered as indices of an absence of multicollinearity between the study variables. Finally, Durbin Watson values were explored to verify the absence of residual problems, and those close to the threshold value of 2 were considered adequate [91]. 

To evaluate the Hypotheses (from H_2_ to H_8_), several serial mediation models with four factors were fit to examine whether the association between Future Anxiety and Psychological Distress (Stress, Anxiety and Depression) was mediated by Intolerance of Uncertainty and Non-Pathological Worry. Model 6 of the PROCESS macro in SPSS [92] has been used to conduct three serial mediation models. In these serial mediation patterns, Stress, Anxiety and Depression were selected as dependent variables (*Y*), Future Anxiety as the independent variable (*X*), Intolerance of Uncertainty and Non-Pathological Worry as Mediators (*M*_1_ and *M*_2_), and Age and Gender were measured as potential covariates, based on literature review and results of correlations and t-tests. In all three serial models, mediation tests were performed simultaneously through two triangle pathways and one quadrangle pathway: Future Anxiety → Intolerance of Uncertainty → Psychological Distress (Ind1); Future Anxiety → Non-Pathological Worry → Psychological Distress (Ind2); Future Anxiety → Intolerance of Uncertainty → Non-Pathological Worry → Psychological Distress (Ind3). The statistical significance of mediation effects was examined using bootstrapping methods to estimate bias-corrected asymmetric confidence intervals (CIs) with 5000 resamples with replacement and verify the robustness of mediating effects (95% CIs not inclusive of zero indicate significant effects). The Sobel test technique based on a normality assumption was also used to further confirm the indirect effect of single mediators (*z* > 1.96; *p* < 0.05). Finally, sensitivity analyses were conducted to detect the effects of the opposing relationship between Intolerance of Uncertainty and Non-Pathological Worry.

## 3. Results

### 3.1. Descriptive Analyses and Group Differences

The mean of the DFS was 19.29 (*SD* = 6.78), that of the IUS-12 was 35.36 (*SD* = 9.81), and that of the WQD was 49.28 (*SD* = 19.45). Those for Stress, Anxiety, and Depression were, respectively, 25.60 (*SD* = 10.04), 17.57 (*SD* = 10.87), and 20.77 (*SD* = 10.78). All variables in this study were normally distributed, with values of Skewness and Kurtosis ranging from +1.5 to −1.5. All descriptive analyses, Cronbach’s *α*, Skewness and Kurtosis are shown in Table 1.

The *t*-tests showed significant gender differences. Female participants reported higher levels than male ones for the DFS (*M*_F_ = 21.10 vs. *M*_M_ = 17.40; *t* (300) = 4.74; *p* < 0.001; *d* = 0.55), the WDQ (*M*_F_ = 52.88 vs. *M*_M_ = 45.55; *t* (300) = 3.19; *p* = 0.002; *d* = 0.18), Stress (*M*_F_ = 28.66 vs. *M*_M_ = 22.40; *t* (300) = 5.36; *p* < 0.001; *d* = 0.65), Anxiety (*M*_F_ = 20.40 vs. *M*_M_ = 17.73; *t* (300) = 4.54; *p* < 0.001; *d* = 0.52) and Depression (*M*_F_ = 22.51 vs. *M*_M_ = 18.97; *t* (300) = 2.85; *p* = 0.005; *d* = 0.34). 

ANOVA and post hoc tests also showed significant differences in relation to occupation. Indeed, students reported higher levels than working students for the DFS (*M*_S_ = 20.08 vs. *M*_WS_ = 17.14; *F* (3, 298) = 4.43; *p* = 0.005; *η*^2^ = 0.04) and for the WDQ (*M*_S_ = 51.64 vs. *M*_WS_ = 43.69; *F* (3, 298) = 6.17; *p* < 0.001; *η*^2^ = 0.06). No further significant group differences were found considering other socio-demographic variables.

### 3.2. Correlation Analyses

Pearson’s correlations between participants’ age, gender, and psychological variables highlighted a positive, significant, and strong correlation between Future Anxiety (DFS) Intolerance of Uncertainty (IUS-12), and Non-Pathological Worry (WDQ). At the same time, Future Anxiety was also positively and significantly associated with Stress, Anxiety and Depression (DASS-21). Age was significantly and negatively correlated with Stress, Anxiety and Depression while Gender was significantly and positively correlated with Future Anxiety, Intolerance of Uncertainty, and all outcomes of Psychological Distress. All correlation results are shown in Table 2.

### 3.3. Preliminary Analyses

Harman’s single-factor test extracted 12 factors with eigenvalues greater than 1. The first factor explained 29.55% of the total variance, which is below the recommended threshold of 50% [93]. This result indicated that common method bias does not hinder this study. 

Multicollinearity diagnostics showed that the tolerance values varied between 0.43 and 0.63, and Variance Inflation Factor (VIF) values ranged from 1.57 to 2.34. Durbin–Watson Values, evaluated for the three models on mental health outcomes, were, respectively 1.89, 2.10, and 2.08. These findings indicated that multicollinearity and residual problems were not present.

### 3.4. Serial Mediation Model on Stress

**Figure 2:**
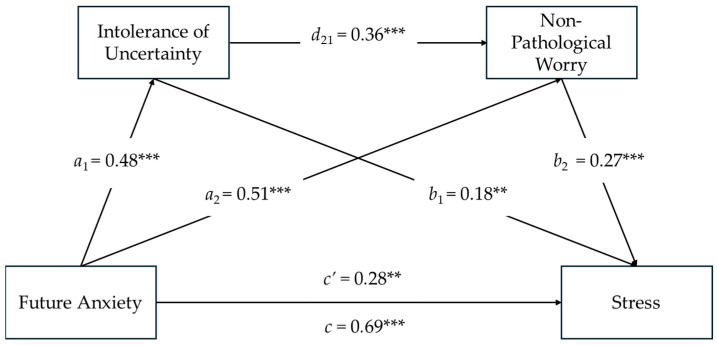
Serial Mediating Effects of Intolerance of Uncertainty and Non-Pathological Worry between Future Anxiety and Stress. Notes: *N* = 302; ** *p* < 0.01; *** *p* < 0.001; All present effects are standardized (see Table S1 in Supplementary Material for unstandardized coefficients); Control Variables: Age, Gender; *a*_1_ = effect of Future Anxiety on Intolerance of Uncertainty; *a*_2_ = effect of Future Anxiety on Non-Pathological Worry; *d*_21_ = effect of Intolerance of Uncertainty on Non-Pathological Worry; *b*_1_ = effect of Intolerance of Uncertainty on Stress; *b*_2_ = effect of Non-Pathological Worry on Stress; *c*’ = direct effect of Future Anxiety on Stress; *c* = total effect of Future Anxiety and Mediators on Stress.

As seen in Figure 2, the results of serial mediation analysis showed that Future Anxiety had a significant direct effect on Intolerance of Uncertainty (*a*_1_ = 0.48; *SE* = 0.08; *t* = 9.07; *p* < 0.001) and on Non-Pathological Worry (*a*_2_ = 0.51; *SE* = 0.13; *t* = 11.53; *p* < 0.001). Similarly, Intolerance of Uncertainty positively predicted Non-Pathological Worry (*d*_21_ = 0.36; *SE* = 0.08; *t* = 8.40; *p* < 0.001). Furthermore, Intolerance of Uncertainty (*b*_1_ = 0.18; *SE* = 0.06; *t* = 3.25; *p* < 0.01) and Non-Pathological Worry (*b*_2_ = 0.27; *SE* = 0.03; *t* = 3.94; *p* < 0.001) had a significant effect on Stress. The co-presence of a significant total effect between Future Anxiety and Stress (*c* = 0.69; *SE* = 0.07; *t* = 9.31; *p* < 0.01; CI [0.55, 0.84]) and, at the same time, a significant direct effect of Future Anxiety on Stress (*c*’ = 0.28; *SE* = 0.09; *t* = 3.01; *p* < 0.01; CI [0.10, 0.47])—despite the addition of the two mediators—was indicative of a partially mediated model.

The total indirect effect (i.e.,) of Future Anxiety through Intolerance of Uncertainty and Non-Pathological Worry on Stress was significant (*total* i.e., =0.28; *SE* = 0.04; 95%; CI [0.18, 0.36]). The indirect effect of Future Anxiety on Stress via Intolerance of Uncertainty was significant (Ind1 = 0.09; *SE* = 0.03; 95%; CI [0.03, 0.14]; Sobel *z* = 3.06; *p* = 0.002), as was the indirect effect of Future Anxiety on Stress via Non-Pathological Worry (Ind2 = 0.14; *SE* = 0.03; 95%; CI [0.07, 0.21]; Sobel *z* = 3.71; *p* = 0.000). As a result of a serial mediating effect, the indirect effect of Future Anxiety on Stress via Intolerance of Uncertainty and Non-Pathological Worry was significant (Ind3 = 0.05; *SE* = 0.01; 95%; CI [0.02, 0.07]).

Regarding the covariates, while age did not have a significant impact on the total effect model (*β*age = −0.06; *p* = 0.22), gender was found to have a statistically significant impact (*β*gender = 0.18; *p* < 0.000). This finding indicates that women experienced more Stress levels about Future Anxiety compared to men and confirms the *t*-test results.

### 3.5. Serial Mediation Model on Anxiety

**Figure 3:**
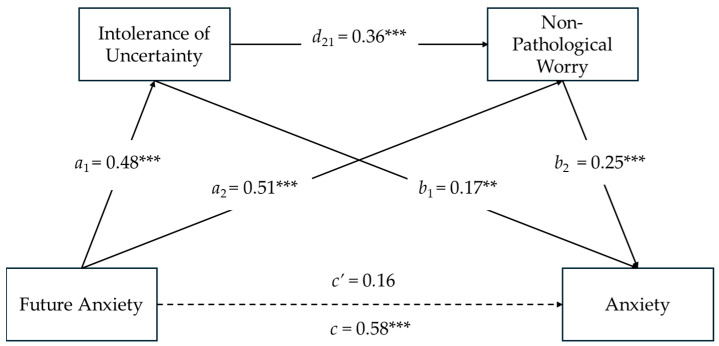
Serial Mediating Effects of Intolerance of Uncertainty and Non-Pathological Worry between Future Anxiety and Anxiety. Notes: *N* = 302; ** *p* < 0.01; *** *p* < 0.001; All present effects are standardized (see Table S1 in Supplementary Material for unstandardized coefficients); Control Variables: Age, Gender; *a*_1_ = effect of Future Anxiety on Intolerance of Uncertainty; *a*_2_ = effect of Future Anxiety on Non-Pathological Worry; *d*_21_ = effect of Intolerance of Uncertainty on Non-Pathological Worry; *b*_1_ = effect of Intolerance of Uncertainty on Anxiety; *b*_2_ = effect of Non-Pathological Worry on Anxiety; *c*’ = direct effect of Future Anxiety on Anxiety (dashed line = non-significant effect); *c* = total effect of Future Anxiety and Mediators on Future Anxiety.

As seen in Figure 3, the results of serial mediation analysis showed that Future Anxiety had a significant direct effect on the mediators, and Intolerance of Uncertainty positively predicted Non-Pathological Worry (see Figure 2 and Model 1). Intolerance of Uncertainty (*b*_1_ = 0.17; *SE* = 0.07; *t* = 2.77; *p* < 0.01) and Non-Pathological Worry (*b*_2_ = 0.25; *SE* = 0.04; *t* = 3.37; *p* < 0.001) had a significant effect on Anxiety. The co-presence of a significant total effect between Future Anxiety and Anxiety (*c* = 0.58; *SE* = 0.09; *t* = 6.65; *p* < 0.001; CI [0.41, 0.75]) and, at the same time, a non-significant direct effect of Future Anxiety on Anxiety (*c*’ = 0.16; *SE* = 0.11; *t* = 1.45; *p* < 0.15; CI [−0.06, 0.38])—despite the addition of the two mediators—was indicative of a fully mediated model.

The total indirect effect of Future Anxiety through Intolerance of Uncertainty and Non-Pathological Worry on Anxiety was significant (*total*, i.e., =0.26; *SE* = 0.05; 95%; CI [0.16, 0.35]). The indirect effect of Future Anxiety on Anxiety via Intolerance of Uncertainty was significant (Ind1 = 0.08; *SE* = 0.03; 95%; CI [0.02, 0.15]; Sobel *z* = 2.65; *p* = 0.008), as was the indirect effect of Future Anxiety on Anxiety via Non-Pathological Worry (Ind2 = 0.13; *SE* = 0.04; 95%; CI [0.06, 0.21]; Sobel *z* = 3.23; *p* = 0.001). As a result of a serial mediating effect, the indirect effect of Future Anxiety on Anxiety via Intolerance of Uncertainty and Non-Pathological Worry was significant (Ind3 = 0.08; SE = 0.01; 95%; CI [0.02, 0.10]).

Regarding the covariates, while age did not have a significant impact on the total effect model (*β*age = −0.08; *p* = 0.12), gender was found to have a statistically significant impact (*β*gender = 0.16; *p* < 0.01). This finding indicates that women experienced more Anxiety levels about Future Anxiety compared to men and, as with the previous model, corroborates the *t*-test findings.

### 3.6. Serial Mediation Model on Depression

**Figure 4:**
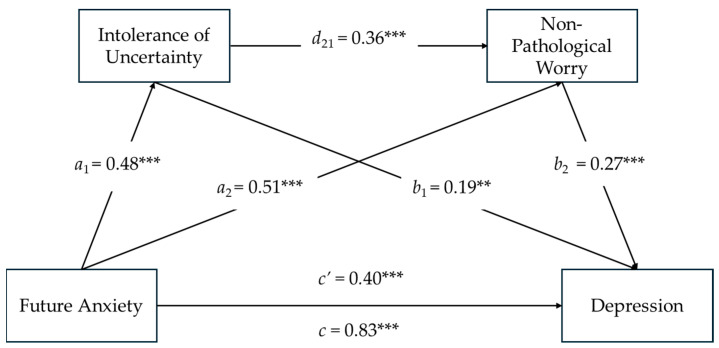
Serial Mediating Effects of Intolerance of Uncertainty and Non-Pathological Worry between Future Anxiety and Depression. Notes: *N* = 302; ** *p* < 0.01; *** *p* < 0.001; All present effects are standardized (see Table S1 in Supplementary Material for unstandardized coefficients); Control Variables: Age, Gender; *a*_1_ = effect of Future Anxiety on Intolerance of Uncertainty; *a*_2_ = effect of Future Anxiety on Non-Pathological Worry; *d*_21_ = effect of Intolerance of Uncertainty on Non-Pathological Worry; *b*_1_ = effect of Intolerance of Uncertainty on Depression; *b*_2_ = effect of Non-Pathological Worry on Depression; *c*’ = direct effect of Future Anxiety on Depression; *c* = total effect of Future Anxiety and Mediators on Depression.

As seen in Figure 4, the results of serial mediation analysis showed that Future Anxiety had a significant direct effect on the mediators, and Intolerance of Uncertainty positively predicted Non-Pathological Worry (see Figure 2 and Model 1). Furthermore, Intolerance of Uncertainty (*b*_1_ = 0.19; *SE* = 0.06; *t* = 3.20; *p* < 0.01) and Non-Pathological Worry (*b*_2_ = 0.27; *SE* = 0.04; *t* = 3.88; *p* < 0.001) had a significant effect on Depression. The co-presence of a significant total effect between Future Anxiety and Depression (*c* = 0.84; *SE* = 0.08; *t* = 10.36; *p* < 0.000; CI [0.68, 0.99]) and, at the same time, a significant direct effect of Future Anxiety on Depression (*c’* = 0.40; *SE* = 0.10; *t* = 3.88; *p* < 0.000; CI [0.20, 0.60])—despite the addition of the two mediators—was indicative of a partially mediated model. 

The total indirect effect of Future Anxiety through Intolerance of Uncertainty and Non-Pathological Worry on Depression was significant (*total*, i.e., =0.27; *SE* = 0.04; 95%; CI [0.19, 0.35]). The indirect effect of Future Anxiety on Depression via Intolerance of Uncertainty was significant (Ind1 = 0.10; *SE* = 0.03; 95%; CI [0.03, 0.15]; Sobel *z* = 3.02; *p* = 0.002), as was the indirect effect of Future Anxiety on Depression via Non-Pathological Worry (Ind2 = 0.14; *SE* = 0.04; 95%; CI [0.06, 0.21]; Sobel *z* = 3.68; *p* = 0.000). As a result of a serial mediating effect, the indirect effect of Future Anxiety on Depression via Intolerance of Uncertainty and Non-Pathological Worry was significant (Ind3 = 0.05; *SE* = 0.01; 95%; CI [0.02, 0.08]).

Regarding the covariates, Age (*β*age = −0.06; *p* = 0.22) and Gender (*β*gender = 0.02; *p* = 0.66) did not have a significant impact on the total effect model, indicating that there were no differences between females and males in Depression levels about Future Anxiety.

### 3.7. Sensitivity Analyses

The sensitivity analysis of serial mediation models is shown in Supplementary Material (Table S2). The opposing direction effects of Intolerance of Uncertainty and Non-Pathological Worry on the association between Future Anxiety and Stress, Anxiety and Depression were presented in the sensitivity analyses (Future Anxiety → Non-Pathological Worry → Intolerance of Uncertainty → Stress, Anxiety, Depression). The variable of Non-Pathological Worry was included as the first mediator, while Intolerance of Uncertainty was considered as the second mediator. According to the sensitivity analysis and by reversing the mediators, we found that the effect of Future Anxiety on Intolerance of Uncertainty was not significant (*a*_2_ = 0.11, *p* = 0.07). At the same time, the indirect mediating effect of Intolerance of Uncertainty in the relationship between Future Anxiety and Stress (Ind2 = 0.02; *SE* = 0.01; CI [−0.005, 0.05]), Anxiety (Ind2 = 0.02; *SE* = 0.01; CI [−0.004, 0.05]) and Depression (Ind2 = 0.02; *SE* = 0.01; CI [−0.004, 0.05]) became not significant. Overall, this inversion reduced the total effect of all three models.

## 4. Discussion

The present study explored the relationship between Future Anxiety, Intolerance of Uncertainty, Non-Pathological Worry and Stress, Anxiety and Depression in a group of young Italian adults. To our knowledge, such an investigation on Italian subjects is unprecedented, probably due to the recent introduction of the construct of ‘Future Anxiety’ [39]. In this investigation, three serial mediation models have been hypothesized and assessed to explore the impact of Future Anxiety on Stress, Anxiety and Depression levels, as well as the indirect effects that Intolerance of Uncertainty and Non-Pathological Worry (used here as joint mediators) have on this relationship. Our results are in line with the ‘youth mental health emergency’ that is characterizing the Italian post-COVID context [26,79], and highlight severe levels of Stress, Anxiety and Depression in the participants, which are particularly heightened in the female group. These results align with those obtained by Van Loo et al. [16], which recently identified an increase in internalizing problems, precisely in young people aged between 18 and 30 (namely the target of this study), but also with recent studies on the effects of COVID-19 on young adults [17,19,21]. Furthermore, they also share similarities with Jannini et al. [39], Awad et al. [46], and Torrado & Garcia-Castro [47], confirming our hypothesis H_1_ and shedding light on the significant direct effect of Future Anxiety on Stress and Depression (H_2_). On the contrary, the results from our Anxiety model differ from previous studies, as Intolerance of Uncertainty and Non-Pathological Worry together completely mediate the impact of Future Anxiety on Anxiety.

If specific contextual factors that affect the representation of young people’s future and hinder the transition to adulthood have long been reported in the literature [7,9,12,14], more recently it has been highlighted that even collective events with traumatic potential—such as the COVID-19 pandemic, wars, and the climate crisis—are fueling the Dark Future time perspective [48,49,50].

We might say that, in addition to specific external factors, the increase in uncertainty, pessimism, and the consequent decrease in motivation to take action found among young Italian adults [38] are also having an impact on the perception of not having enough resources to face difficulties, viz. on the internal factor of Future Anxiety highlighted by Jannini et al. [39].

Considering the three serial mediation models on Stress, Anxiety and Depression conducted, the results confirm our hypotheses H_3_ and H_4_, suggesting that the higher the level of Future Anxiety, the more the probability of experiencing greater levels of Intolerance of Uncertainty and Non-Pathological Worry increases. We believe that the strong associations found in our report between these psychological variables (see Table 2), which had not yet been investigated in Italy before, are of significant importance for better understanding the contours of the widely reported psychological distress in young adults, and the risk factors that are triggering it.

At an age where instability is a distinctive trait [2], imagining the future requires tolerating moments of uncertainty and fear of the unknown. It is in these moments that the creative drive to explore life alternatives arises and allows the definition of new development trajectories in the construction of an authentic and coherent life plan [94] in different fields and starting from the objectives we intend to pursue [95]. From this perspective, Intolerance of Uncertainty appears to be an obstacle to this exploratory capacity, a risk factor that can alter the perception of the present and the future, fueling fears, anxieties, worries, and an anguished attitude towards the future in which an illusory desire for control can be activated as a defense [51,52,53]. 

Our results also highlight the relationship between Future Anxiety and Non-Pathological Worry, confirming the link between these variables and providing an empirical basis for the definition of the Dark Future time perspective as “a specific inclination to think about the future with worry” [39] (p. 86). In fact, they suggest that higher levels of Future Anxiety correspond to a higher likelihood of experiencing daily difficulties and stressors with great worry (e.g., economic autonomy, difficulties in the world of work, relational problems, and uncertainty about the future), which impacts the psychological well-being of young people. Furthermore, our results confirmed previous studies according to which the future perception of threatening and/or uncertain situations—which we explored through a similar construct, i.e., Future Anxiety—plays a pivotal role in the Worry cognitive process [68,69].

In continuity with this, findings of mediation analyses show that both Intolerance of Uncertainty (see Ind1) and Non-Pathological Worry (see Ind2) are significant mediators in the relationship between Future Anxiety and Stress, Anxiety and Depression. Regarding the first mediator, our results suggest that the relationship between Future Anxiety and Psychological Distress could be mediated by the dispositional tendency to tolerate states of uncertainty (H_5_), in line with the study by Freeston et al. [52] on “Uncertainty Distress Model”. At the same time, our results contribute to further explore the relation between vision about the future and intolerance of uncertainty, which has already been partly reported in the past [96], highlighting how an anxious attitude towards the future—strongly influenced by both personal characteristics and contextual factors [41]—can increase the likelihood of experiencing states of uncertainty experienced as threatening.

Regardless of the type of stressor, subjects who do not tolerate uncertainty or who experience it negatively are more at risk of experiencing forms of psychological distress, especially in the internalizing sphere [55]. Not only that, as revealed more recently, Intolerance of Uncertainty can also fuel the impact of collective traumatic events on psychological distress [49,56]. 

In all three mediation models explored, Non-Pathological Worry was also a significant mediator in the relationship between Future Anxiety and Stress, Anxiety and Depression (H_6_). This relationship had not yet been investigated in the Italian context, despite several reports stating an increase in pervasive and varied worries in young adults [37,79]. In line with Kelly et al. [65,66], our results highlight that non-pathological worries are fueling Stress, Anxiety and Depression, acquiring a maladaptive function that fuels youthful malaise. At the same time, findings show that a negative attitude toward the future increases the likelihood of experiencing the typical difficulties of the transition to adulthood (e.g., work, economy, relationship) with greater worry [39] and that this—as found in previous studies [67,68]—increases the risk of experiencing Stress, Anxiety and Depression. As a starting point for future research, we wonder whether and to what extent the increase in daily worries and their impact on mental health are also connected to specific sources of contemporary malaise, including excessive pressure for perfection, urgency, performance, and competition [97,98].

Through the serial mediation models for Stress, Anxiety and Depression, our results also confirm the joint mediation effect of Intolerance of Uncertainty and Non-Pathological Worry (H_7_). In line with the contributions by Freeston et al. [70] and with the Intolerance of Uncertainty Model (IUM) [71], they emphasize that Intolerance of Uncertainty functions as a dispositional characteristic profoundly associated with Worry and, specifically, a risk factor that can predict greater levels of worry in young Italian adults. These results, confirmed by subsequent sensitivity analyses, agree with the studies by Koerner and Dugas [72] and confirm Intolerance of Uncertainty as a factor of cognitive vulnerability to Worry. Although both mediators, even if taken individually, were found to be risk factors for psychological well-being, it seems to us that the serial models offer an overall vision of the relationship between the variables investigated, providing a clearer understanding of the relation between Intolerance of Uncertainty and Non-Pathological Worry and their joint mediating effect on Future Anxiety and Psychological Distress (i.e., Stress, Anxiety and Depression). The significant serial link (see Ind3) integrates several factors that impact the relationship between Future Anxiety and Psychological Distress and offers a way to better understand why subjects with greater Future Anxiety also tend to have greater levels of Psychological Distress. Without the aim of saturating the field of investigation, these data could open new avenues in understanding the interaction between Future Anxiety and Psychological Distress, providing new food for thought to the psychological literature that is interested in these constructs. 

To conclude, our results also highlighted greater psychological fragility in women, confirming the hypothesis we formulated based on the reference literature (H_8_). T-test analyses showed significantly higher levels of Stress, Anxiety and Depression, Future Anxiety and Non-pathological worry in women when compared to male participants. The results are also partially confirmed in serial mediation models in which women reported higher levels of Stress and Anxiety in relation to Future Anxiety. The sociocultural framework has highlighted how the type of upbringing imparted to women from an early age may play a pivotal role in the gender difference regarding internalizing symptoms since superior social cognition and a greater ability to attune to others, central functions in raising progeny [99] appear to support a greater sensitivity to rejection, separation and criticism, all of which have been found to be central characteristics in anxiety and depression [100,101]. At the same time, however, there are studies that have shed light on how gender difference in the problems of the internalizing sphere can be influenced by biological and hormonal factors associated with typically female experiences—such as the menstrual cycle, pregnancy and menopause—that seem to play a relevant role in both the emergence of affective disorders and their exacerbation [102].

Although there are several approaches in the literature that attempt to explain gender difference in affective disorders, our results are in line with studies reporting the greater risk of psychological distress in the internalizing sphere of women [103,104]. Furthermore, these findings reiterate those that emerged in studies on mental health concerning the COVID-19 pandemic [18,105,106,107] and the most recent data reported by Van Loo et al. [16], according to which the increase in internalizing problems in the target group to which this study refers particularly concerns women.

### Limitations and Directions for Future Research

First of all, the limitations of the present study concern the web-based convenience sampling methodology, which implies specific biases such as the volunteer bias, related to the specific characteristics of subjects who voluntarily participate in a study. Another bias concerns the use of a mono-method, as the assessment of all variables using self-report instruments may have caused inflation in the observed associations. In addition, this is a cross-sectional study that does not allow for the establishment of causal/directional relationships among the variables explored, which future research could overcome by adopting a multi-method approach. Finally, the participants are mostly young adult students who are predominantly from southern Italy, thus future studies could make up for this shortcoming by considering a more diversified sample (for instance, including more young working adults and/or young adults who come from different Italian regions). Overall, the highlighted limitations do not allow the results obtained to be generalized to the entire population of young Italian adults, so we suggest that prospective research consider increasing the representativeness of the sample in the future.

## 5. Conclusions and Practical Implications

The present study investigated the indirect psychological impact of Intolerance of Uncertainty and Non-Pathological Worry on Future Anxiety and Italian young adults’ psychological distress. This is, to our knowledge, the first Italian study to explore mediating variables in the relationship between Future Anxiety and mental health, highlighting the joint mediating effect that Intolerance of Uncertainty and Non-Pathological Worry have in this relationship.

We believe that our research contributes to enriching innovatively the psychological literature that has long explored the connection between future perspective and youth mental health, delving into the contours of the ‘youth mental health emergency’ that is being talked about in Italy [26]. Furthermore, in our opinion, highlighting how Intolerance of Uncertainty and non-pathological daily worries constitute significant risk factors that increase the impact of Future Anxiety on the mental well-being of young adults offers important insights on how to design more targeted intervention projects aimed at supporting young people’s ability to deal more effectively with the developmental tasks of their transition to adulthood. Our results stress the importance of working on the implementation of tolerance of uncertainty connected not only to the age-specific tasks and difficulties encountered today but also to collective events with traumatic potential. Although Intolerance of Uncertainty is a dispositional factor, previous literature suggests that it is particularly influenced by external factors such as the amount and quality of information and tools available for understanding and managing a given event/difficulty, which can fuel or reduce uncertainty [54]. Without denying the importance of the reality on which daily worries and the uncertainty connected to them are grounded, we maintain that classifying them in the physiological tasks of the evolutionary transition, sharing them and, at the same time, receiving support to reappropriate oneself of the chance of having a positive impact in the world and on one’s future are important elements for a successful individual and group empowerment intervention. It is in the group space guided by an expert that the narration and sharing of worries, anxieties, uncertainties, and negative emotions can be represented and understood, an effort that is known to reduce their negative impact and support psychological well-being [108].

In our opinion, some things are pivotal in rediscovering a network of connections, reducing the sense of impotence and loneliness, and rekindling the hope of being able to co-build possible resources and alternatives together. These include the promotion of uncertainty as a growth factor that is not only physiological but also positive to the extent that its tolerance opens the way to the individual and creative exploration of one’s growth path, and the support to address shared and common concerns and rediscover oneself as an ‘agentive participant’ in the world [109]. The mediating role that Intolerance of Uncertainty and daily worries play on the impact that Future Anxiety has on Psychological Distress implies that working on the former could have a positive impact on Future Anxiety and, consequently, on the negative role that it performs on the mental well-being of young Italian adults.

## Figures and Tables

**Table 1:** Means, Standard Deviations, Cronbach’s α, Skewness and Kurtosis.

	Males (*N* = 148)		Females (*N* = 154)		Total Sample (*N* = 302)					
	*M*	*SD*	*M*	*SD*	*M*	*SD*	*Min-Max*	*a*	*Skew.*	*Kurt.*
DFS	17.40	7.28	21.10	5.73	19.29	6.78	0–30	0.88	−0.67	0.19
IUS-12	34.32	9.31	36.36	10.20	35.36	9.81	15–60	0.88	0.24	−0.43
WDQ	45.55	19.94	52.88	18.32	49.29	19.45	3–96	0.92	−0.05	−0.51
STRESS	22.40	9.62	28.66	9.48	25.60	10.04	0–42	0.82	−0.19	−0.68
ANXIETY	14.72	9.60	20.32	11.33	17.58	10.87	0–42	0.88	0.26	−0.75
DEPRES.	18.97	10.47	22.51	10.84	20.77	10.79	0–42	0.88	0.07	−0.83

Notes: DFS: Dark Future Scale; IUS-12: Intolerance of Uncertainty Scale; WDQ: Worry Domains Questionnaire; Stress, Anxiety and Depression: dimensions of DASS-21.

**Table 2:** Correlations between participants’ age and gender and psychological variables.

	1	2	3	4	5	6	7	8
1. Age	--							
2. Gender	--	--						
3. DFS	−0.09	0.27 **	--					
4. IUS-12	0.03	0.10	0.47 **	--				
5. WDQ	−0.09	0.19 **	0.69 **	0.60 **	--			
6. STRESS	−0.11	0.31 **	0.52 **	0.45 **	0.58 **	--		
7. ANXIETY	−0.12 *	0.26 **	0.41 **	0.39 **	0.47 **	0.76 **	--	
8. DEPRESSION	−0.10	0.16 **	0.54 **	0.47 **	0.57 **	0.76 **	0.72 **	--

Notes: *N* = 302; * *p* < 0.05; ** *p* < 0.01.

## Data Availability

The data that support the findings of this study are available from the corresponding author (B.D.R.), upon reasonable request.

## Supplementary Materials

The following supporting information can be downloaded at: https://www.mdpi.com/article/10.3390/ejihpe14060121/s1, Table S1: Total models summary with standardized and unstandardized coefficients. Table S2: Sensitivity analyses with mediators’ inversion.
