# Supplementary material for: Serial Mediation Models of Future Anxiety and Italian Young Adults Psychological Distress: The Role of Intolerance of Uncertainty and Non-Pathological Worry
Source: Eur J Investig Health Psychol Educ. 2024 Jun 20;14(6):1834–52. doi: 10.3390/ejihpe14060121 (PMC11202537; doi:10.3390/ejihpe14060121)
Supplement: Supplementary file 1 [file ejihpe-14-00121-s001.zip › ejihpe-3029306-supplementary.pdf]

Supplementary Materials

Results of Serial mediation model on Stress, Anxiety and Depression

Table S1. Total models summary with standardized and unstandardized coefficients

	Unstandardized estimates				Standardized estimates			
	**Coeff.**	**SE**	**LLCI**	**ULCI**	**Coeff.**	**BootSE**	**LLCI**	**ULCI**
**Mediation Model 1 = Stress**								
Total Effect	0.69	0.07	0.55	0.84				
Direct Effect	0.28	0.09	0.10	0.47				
DFS → IU	0.70	0.08	0.54	0.85	0.48***			
DFS → WDQ	1.47	0.13	1.22	1.72	0.51***			
IU → WDQ	0.71	0.08	0.55	0.88	0.36***			
IU → STRESS	0.19	0.06	0.07	0.30	0.18**			
WDQ → STRESS	0.14	0.03	0.07	0.21	0.27***			
Total indirect effect	0.41	0.06	0.27	0.53	0.28	0.04	0.18	0.36
Ind1	0.13	0.04	0.05	0.21	0.09	0.03	0.03	0.14
Ind2	0.21	0.05	0.10	0.31	0.14	0.03	0.07	0.21
Ind3	0.07	0.02	0.03	0.11	0.05	0.01	0.02	0.07
**Total Stress Model Summary: R^2 = 0.31; $F_{(3,298)}$ = 44.08; p < 0.000**								
**Mediation Model 2 = Anxiety**								
Total Effect	0.58	0.09	0.41	0.75				
Direct Effect	0.16	0.11	-0.06	0.38				
DFS → IU	0.70	0.08	0.54	0.85	0.48***			
DFS → WDQ	1.47	0.13	1.22	1.72	0.51***			
IU → WDQ	0.71	0.08	0.55	0.88	0.36***			
IU → ANXIETY	0.19	0.07	0.05	0.33	0.17**			
WDQ → ANXIETY	0.14	0.04	0.06	0.22	0.25***			
Total indirect effect	0.41	0.08	0.26	0.57	0.26	0.05	0.16	0.35
Ind1	0.13	0.05	0.03	0.24	0.08	0.03	0.02	0.15
Ind2	0.21	0.06	0.09	0.34	0.13	0.04	0.06	0.21
Ind3	0.07	0.02	0.03	0.12	0.08	0.01	0.02	0.10
**Total Anxiety Model Summary: R^2 = 0.20; $F_{(3,298)}$ = 24.21; p < 0.000**								
**Mediation Model 3 = Depression**								
Total Effect	0.84	0.08	0.68	0.99				
Direct Effect	0.40	0.10	0.20	0.60				
DFS → IU	0.70	0.08	0.54	0.85	0.48***			
DFS → WDQ	1.47	0.13	1.22	1.72	0.51***			
IU → WDQ	0.71	0.08	0.55	0.88	0.36***			
IU → DEPRESSION	0.20	0.06	0.08	0.33	0.19**			
WDQ → DEPRESSION	0.15	0.04	0.07	0.23	0.27***			

Total indirect effect	0.43	0.07	0.30	0.58	0.27	0.04	0.19	0.35
Ind1	0.14	0.05	0.05	0.24	0.10	0.03	0.03	0.15
Ind2	0.22	0.06	0.10	0.34	0.14	0.04	0.06	0.21
Ind3	0.07	0.02	0.03	0.12	0.05	0.01	0.02	0.08
**Total Depression Model Summary:** R^2 **= 0.30;** $F_{(3,298)}$ **= 40.69;** $p < 0.000$								

Note: * $p < 0.05$ ** $p < 0.01$; *** $p < 0.001$; Ind 1: Future Anxiety → Intolerance of Uncertainty → Psychological Distress; Ind2: Future Anxiety → Non-Pathological Worry → Psychological Distress; Ind3: Future Anxiety → Intolerance of Uncertainty → Non-Pathological Worry → Psychological Distress. For indirect effects: significance is given by the absence of zeros in the confidence intervals.

Table S2. Sensitivity analyses with mediators' inversion

	**Unstandardized estimates**				**Standardized estimates**			
	**Coeff.**	**SE**	**LLCI**	**ULCI**	**Coeff.**	**BootSE**	**LLCI**	**ULCI**
**Mediation Model 1 = Stress**								
Total Effect	0.67	0.07	0.53	0.82				
Direct Effect	0.27	0.09	0.08	0.45				
DFS → WDQ	1.97	0.13	1.72	2.22	0.68***			
DFS → IU	0.16	0.09	-0.02	0.35	0.11			
WDQ → IU	0.26	0.03	0.20	0.32	0.41***			
WDQ → STRESS	0.14	0.03	0.07	0.21	0.28***			
IU → STRESS	0.18	0.06	0.06	0.29	0.17**			
Total indirect effect	0.41	0.04	0.28	0.53	0.27	0.04	0.18	0.36
Ind1	0.28	0.07	0.10	0.41	0.19	0.04	0.10	0.28
Ind2	0.03	0.02	-0.01	0.07	0.02	0.01	-0.05	0.05
Ind3	0.06	0.02	0.02	0.11	0.06	0.02	0.02	0.11
**Mediation Model 2 = Anxiety**								
Total Effect	0.55	0.09	0.38	0.73				
Direct Effect	0.13	0.11	-0.09	0.48				
DFS → WDQ	1.97	0.13	1.72	2.22	0.68***			
DFS → IU	0.16	0.09	-0.02	0.35	0.11			
WDQ → IU	0.26	0.03	0.20	0.32	0.41***			
WDQ → ANXIETY	0.16	0.07	0.08	0.24	0.29**			
IU → ANXIETY	0.16	0.07	0.02	0.30	0.14*			
Total indirect effect	0.26	0.05	0.28	0.58	0.26	0.05	0.17	0.35
Ind1	0.32	0.08	0.15	0.48	0.19	0.05	0.10	0.30
Ind2	0.02	0.02	-0.00	0.05	0.02	0.01	-0.004	0.05
Ind3	0.05	0.02	0.00	0.10	0.05	0.02	0.003	0.10
**Mediation Model 3 = Depression**								
Total Effect	0.81	0.08	0.65	0.97				
Direct Effect	0.37	0.10	0.16	0.57				

DFS → WDQ	1.97	0.13	1.72	2.22	0.68***			
DFS → IU	0.16	0.09	-0.02	0.35	0.11			
WDQ → IU	0.26	0.03	0.20	0.32	0.41***			
WDQ → DEPRESSION	0.16	0.04	0.09	0.24	0.30***			
IU → DEPRESSION	0.17	0.06	0.05	0.30	0.16**			
Total indirect effect	0.44	0.07	0.30	0.57	0.28	0.04	0.19	0.36
Ind1	0.32	0.08	0.17	0.48	0.20	0.05	0.11	0.30
Ind2	0.03	0.02	-0.01	0.08	0.02	0.01	-0.004	0.05
Ind3	0.09	0.04	0.02	0.16	0.06	0.02	0.01	0.10

Note: *$p < 0.05$; **$p < 0.01$; ***$p < 0.001$; Ind 1: Future Anxiety → Non-Pathological Worry → Psychological Distress; Ind2: Future Anxiety → Intolerance of Uncertainty → Psychological Distress; Ind3: Future Anxiety → Non-Pathological Worry → Intolerance of Uncertainty → Psychological Distress. For indirect effects: significance is given by the absence of zeros in the confidence intervals.
